# Differences in the Force Velocity Mechanical Profile and the Effectiveness of Force Application During Sprint-Acceleration Between Sprinters and Hurdlers

**DOI:** 10.3389/fspor.2019.00026

**Published:** 2019-09-12

**Authors:** Ioannis Stavridis, Ilias Smilios, Angela Tsopanidou, Theodosia Economou, Giorgos Paradisis

**Affiliations:** ^1^School of Physical Education and Sport Science, National and Kapodistrian University of Athens, Athens, Greece; ^2^School of Physical Education and Sport Science, Democritus University of Thrace, Komotini, Greece

**Keywords:** force-velocity profile, ratio of force application, sprint performance, sprint mechanics, biomechanics of hurdling

## Abstract

This cross-sectional study aimed to compare the horizontal and vertical force-velocity profile between female sprinters and hurdlers. Twelve high-level athletes (6 sprinters and 6 hurdlers) participated in this investigation. The testing procedures consisted of two maximal 40-m sprints and five to six vertical jumps with additional loads. For the sprint-acceleration performance, the velocity-time data, recorded by a high-speed camera, was used to calculate the variables of the horizontal F-V profile (theoretical maximal values of force [*HZT-F*_0_], velocity [*HZT-V*_0_], power [*HZT-Pmax*], the proportion of the theoretical maximal effectiveness of force application in the antero-posterior direction [*RFmax*], and the rate of decrease in the ratio of horizontal force [*DRF*]). The best trial of each vertical jumping condition, obtained by an optical measurement system, was used to determine the components of the vertical F-V profile (theoretical maximal values of force [*VTC-F*_0_], velocity [*VTC-V*_0_], and power [*VTC-Pmax*]). The female sprinters showed higher statistical differences for *HZT-Pmax* (2.46 ± 0.67, *d* = 2.1, *p* = 0.004), *HZT-V*_0_ (0.45 ± 0.18, *d* = 1.4, *p* = 0.03), and *RFmax%* (2.9 ± 0.9%, *d* = 1.8, *p* = 0.01) than female hurdlers. No statistical differences were observed for *HZT-F*_0_ (0.69 ± 0.3, *d* = 1.15, *p* = 0.07), *DRF%* (−0.24 ± 0.4%, *d* = 0.3, *p* = 0.62), *VTC-F*_0_ (−2.1 ± 3.8, *d* = 0.3, *p* = 0.59), *VTC-V*_0_ (0.25 ± 0.31, *d* = 0.5, *p* = 0.45), and *VTC-Pmax* (1.75 ± 2.5, *d* = 0.4, *p* = 0.5). Female sprinters are able to apply higher horizontally-oriented forces onto the ground during the acceleration phase than female hurdlers.

## Introduction

Sprinting is a cyclic locomotion depended on the mechanical forces produced through the action of the neuromuscular system. During sprint running, the lower limbs have to produce high forces in order to accelerate, and sustain high running speeds (Bret et al., 2002). The purpose of sprint running performance is to cover a required distance in the shortest time. On the other side, the purpose of sprint-hurdle running performance is to successfully cover a required distance in as short time as possible while clearing barriers. In both events, during the acceleration phase, athletes try to generate high levels of horizontal ground-reaction force (GRF), and apply it with effectiveness onto the ground, despite increasing velocity (Morin et al., 2011). A new macroscopic inverse dynamics approach, based on kinematics and kinetics parameters of the runner's body center of mass during sprint-acceleration (horizontal profile) and ballistic push-off movements (vertical profile), can determinate the force-velocity (F-V), and power-velocity (P-V) relationships, and the mechanical effectiveness of force application parameters (Morin and Samozino, 2016). The horizontal force-velocity-power (F-V-P) profile is described by the theoretical maximal values of force (*HZT-F*_0_), velocity (*HZT-V*_0_), and power (*HZT-Pmax*), the proportion of the theoretical maximal effectiveness of force application in the anterior-posterior direction (*RFmax in %*) and the rate of decrease in the ratio of horizontal force as the velocity increases over the entire acceleration phase (*DRF in % s*·*m*^−1^). The vertical F-V-P profile corresponds with the theoretical maximal values of force (*VTC-F*_0_), velocity *(VTC-V*_0_*)*, and power (*VTC-Pmax*). The horizontal and vertical profiles allow to accurately evaluate force, velocity and power developed by lower limbs during sprint running acceleration and loaded squat jumps (SJ) (Morin and Samozino, 2016). Both horizontal and vertical F-V-P profile could provide a deeper insight into the maximal mechanical properties and function of the lower-body muscles. The horizontal F-V-P profile provides information for the specific sprint-acceleration motion, while the vertical F-V-P profile provides information for the maximal levels of force, and velocity of the neuromuscular system (Morin and Samozino, 2016). The F-V-P profile is able to distinguish differences in the mechanical properties of athletes from different sports, levels of practice, playing positions, age, and sex (Buchheit et al., 2014; Cross et al., 2015; Slawinski et al., 2017; Alcazar et al., 2018; Jiménez-Reyes et al., 2018a,b; Haugen et al., 2019).

It is known that high-level sprinters are able to apply high forward-oriented forces onto the supporting ground during the acceleration phase (Weyand et al., 2000; Kugler and Janshen, 2010; Morin et al., 2011; Otsuka et al., 2014; Nagahara et al., 2018). This ability seems to be more important for sprint performance than the total amount of force they are able to produce (Morin et al., 2011). The horizontal component of the GRF and the mechanical effectiveness of force application were significantly correlated to 100-m performance from non-specialists to elite sprinters (Morin et al., 2012). The higher amount of horizontal GRF in sprint-acceleration performance depends on the higher activation and the torque production capability of the hip extensors muscles (Morin et al., 2015). High-level sprinters should produce high amount of force in the anterior-posterior direction and minimize forces in all other directions (lateral and vertical) in order to reach the maximum velocity. If the total horizontal force created during ground contact is positive, horizontal velocity increases. As sprinters reach their maximal velocity, the ground contact duration gets shorter at each step, and the overall orientation gets more vertically-oriented, which is related to an overall progressive vertical orientation of the GRF vector at each support phase (Nagahara et al., 2018). In the hurdle race the athletes must produce great amount of horizontal velocity and maintain it while approaching, and clearing the barriers and running between them. The maximal horizontal velocity that a hurdler can produce depends on the amount of effective force that he can apply during ground contact, throughout the race (Čoh and Iskra, 2012). It would be interesting to examine the mechanical characteristics of top national athletes from the two events where speed is the requirement but with different technical characteristics (i.e., differences in trunk angle during the acceleration phase and maximum speed phase decides hurdle clearance). Would high-level sprinters show a different vertical and horizontal mechanical profile from top high-level hurdlers? It has been proposed that the vertical profile could provide information regarding the maximal level of force and velocity of the athlete's neuromuscular system, whereas horizontal profiling could provide information as to the specific sprint acceleration motion and especially the ability to effectively apply force during sprinting (Morin and Samozino, 2016; Jiménez-Reyes et al., 2018b). For this purpose, the horizontal and vertical force-velocity-power (F-V-P) profile between female sprinters and hurdlers were compared. We hypothesized that sprinters would present higher overall mechanical output capabilities *(HTZ-Pmax)* in the forward direction, higher ability to develop horizontal force at high velocities *(HTZ-V*_0_*)*, higher ability to produce horizontal force during sprint-acceleration *(HTZ-F*_0_*)*, and greater maximal effectiveness of force application *(RFmax)*, than hurdlers.

## Materials and Methods

### Participants

12 high-level female athletes, 6 sprinters (Mean ± SD: age 23.5 ± 3.0 years; stature 1.67 ± 0.07 m; weight 60.1 ± 2.0 kg; personal best in 100-m sprint running performance 11.76 ± 0.2 s), and 6 hurdlers (age 21.0 ± 5.1 years; stature 1.68 ± 0.05 m; weight 59.2 ± 4.6 kg; personal best in 100-m hurdles running performance 14.06 ± 0.3 s) who participated in the finals of their events during the national championship, gave their written informed consent to participate in this study, which was approved by the local ethical committee, and conducted in accordance with the Declaration of Helsinki. No participants reported physical limitations, health problems or musculoskeletal injuries that could compromise testing. Participants were required to refrain from vigorous exercise for 2 days before testing. The tests were conducted over 2 different testing sessions, within the same week, in an indoor stadium and during the competitive athletes' period.

### Testing Procedure

All sessions began with a specific sprint warm-up that involved a 10 min of low-pace running, followed by 5 min of lower limb muscle dynamic stretching, 5 min of sprint-specific drills, and three progressive 40-m sprints separated by 2 min of passive rest (Jiménez-Reyes et al., 2018a). At the first testing session, each athlete performed two maximal sprints of 40-m from a three-point crouching position with 5 min of rest between each trial. The velocity-time data of each sprint was recorded by a high-speed camera (Casio Exilim EX-F1, Tokyo, Japan) sampling at 300 Hz. The high-speed camera was fixed on a tripod, 10-m away from the runway at the half of sprinting distance (i.e., 20-m) and at a height of 1-m corresponding approximately to the height of athlete's center of mass. The video parallax error was corrected to ensure the different split times are measured properly when athletes cross the different targeted distances (5, 10, 15, 20, 25, 30, 35, and 40-m) (Romero-Franco et al., 2017).

At the second testing session, the push-off distance was calculated as the difference between lower limb length (distance from great trochanter to tip of the toes with extended lower limps) and starting height at the squat jump (vertical distance from greater trochanter to ground). Each subject performed vertical maximal SJ without loads and with progressively increasing, five to six, extra loads ranging from 10 to 60 kg. The starting position was self-selected by the participants before the trial and was kept fixed for the subsequent trials using a marker on the squat cage to maintain the same squat depth throughout the experiment (Giroux et al., 2015). The participants were asked to maintain their starting position for about 1–2 s and then apply force as fast as possible and jump for maximum height. Participants kept their arms on their hips for jumps without load and on the bar for loaded jumps. Two valid trials were performed with each load with 3 min of recovery between trials. Jump heights were obtained by using an optical measurement system (OptoJump Next Microgate, Bolzano, Italy).

### Data Processing

The sprint velocity-time video data was analyzed by Kinovea (v.0.8.15) and the best trial was used to determine the components of the horizontal mechanical F-V profile (*HZT-F*_0_*, HZT-V*_0_*, HZT-Pmax, RFmax, DRF*). The acceleration of the athlete's center of mass to the antero-posterior direction can be calculated from the changes in running velocity over time and net horizontal GRF can be calculated by considering the body's center of mass of the athlete and aerodynamic friction of force (Samozino et al., 2016). The entire force-velocity relationship represents the maximal theoretical horizontal force that the lower limbs could produce over one contact at a null velocity (*HZT-F*_0_) and the theoretical maximum velocity that could be produced during a support phase in the absence of mechanical constraints (*HTZ-V*_0_). These variables were calculated as extrapolated from the linear sprint F–V relationship, as the intercept of the x-(force) and y-(velocity) axis of the linear regressions. Multiplying horizontal F and V values for each support phase, the equivalent of maximal mechanical power output *(HTZ-Pmax)* in the forward direction is obtained and computed as *Pmax* = *F*_0_ × *V*_0_*/4*. The ratio of force *(RF)* was calculated as the ratio of the horizontally-oriented component to the total GRF, computed as *RF* = *FHzt/Ftot*. The Rate of decrease in *RF* (*DRF*) computed as the slope of the linear RF–V relationship, as the velocity increases until the end of the acceleration. The parameters derived with this method have been validated compared to force plate measurements and a low absolute bias (≤6%) was found while a high reliability (coefficients of variation (CV) and standard errors of measurement <5%) was observed as well (Samozino et al., 2016; Morin et al., 2019). For the vertical F-V profile, the best trial of each jumping condition was used to determine the components of the mechanical F-V profile *(VTC-F*_0_*. VTC-V*_0_*, VTC-Pmax)*, according to the Samozino's method. This method is based on the fundamental principles of dynamics applied to the body center of mass during a vertical jump and on the analysis of its mechanical energy at different specific instants of the movement (Samozino et al., 2008). The force-axis intercept of the F-V relationship (*VTC-F*_0_) represents the maximal external force lower limbs could produce during a theoretical extension movement at null velocity. The velocity-axis intercept (*VTC-V*_0_) corresponds to the maximal velocity at which lower limbs could extend during a theoretical extension under zero load. The apex of the P-V relationships *(VTC-Pmax)* is the maximal power output lower limbs can produce over one extension and computed as *Pmax* = *F*_0_ × *V*_0_*/4* (Samozino et al., 2013; Jaric, 2016; Morin and Samozino, 2016). A high reliability (ICCs: 0.96–0.99 and CVs: 2.7–8.4%) and validity (absolute bias < 3%, Pearson correlation coefficients: 0.88–0.98, CVs: 4–15%) of this method compared to force plate measurements for the estimation of force, velocity and power during jumping trials has been reported (Samozino et al., 2008; Giroux et al., 2015).

### Statistical Analysis

Data are presented as Means ± standard deviation (SD). Normality (Shapiro-Wilk test) and homogeneity of variance (Levene test) were checked before analyses. Independent samples *t*-tests were used to compare the horizontal (*HTZ-F*_0_*, HTZ-V*_0_*, HTZ-Pmax, RFmax, DRF*), and the vertical (*VTC-F*_0_*, VTC-V*_0_*, VTC-Pmax*) mechanical F-V-P profiles between sprinters, and hurdlers. The magnitude of the differences was also expressed as a standardized mean difference with the corresponding 95% confidence interval. The criteria to interpret the magnitude of the ES [Cohen's d effect size [ES]] was as follows: small (*d* = 0.2), medium (*d* = 0.5), and large (*d* ≥ 0.8) (Cohen, 2013). All statistical analyses were performed using the software package SPSS (IBM SPSS version 25.0, Chicago, IL, USA), and statistical significance was set at an alpha level of 0.05.

## Results

**Figure 1:**
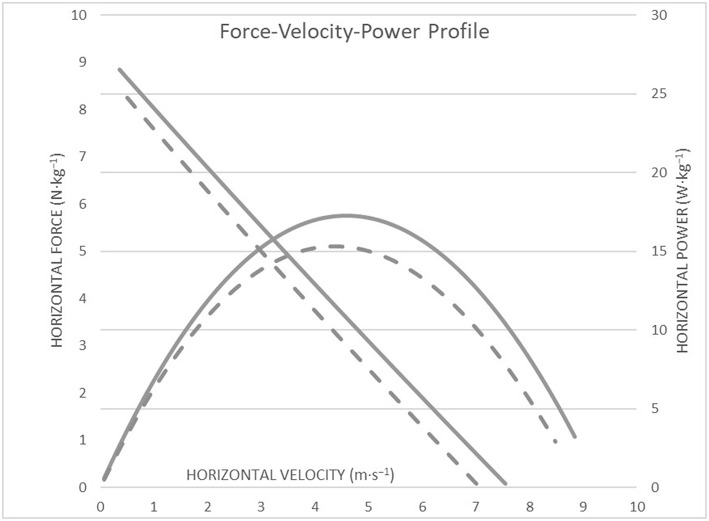
Graphic representation of the relationship between force-velocity and power-velocity as profiled from a 40-m sprint testing procedure between high-level female sprinters (black line) and hurdlers (dashed line). HZT-F_0_ and HZT-V_0_ represent the y and x intercepts of the linear regression, and the theoretical maximum of force, and velocity able to be produced in the absence of their opposing unit. HZT-Pmax represents the maximum power produced, determined as the peak of the polynomial fit between power and velocity.

**Figure 2:**
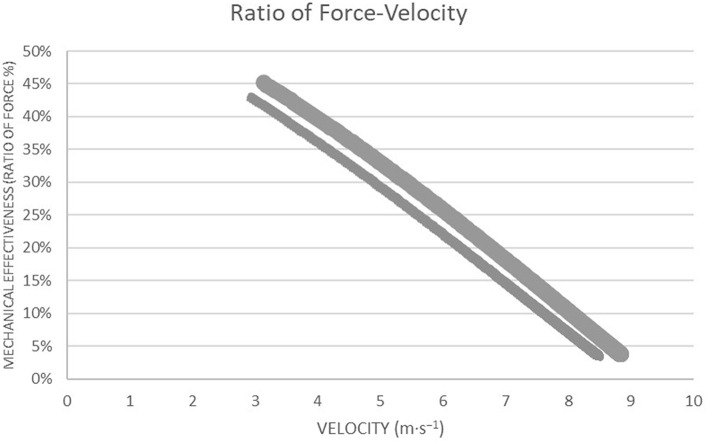
Graphic representation of the Ratio of Force as a function of running velocity during a sprint testing procedure for high-level female sprinters (weighted line) and hurdlers (thin line) and the decrease in the Ratio of Force as velocity increases.

**Figure 3:**
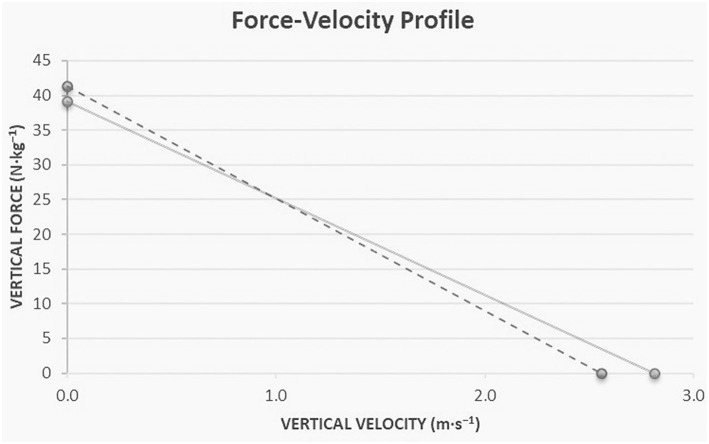
Graphic representation of the relationship between force-velocity as profiled from the vertical jumps with additional loads testing procedure between high-level female sprinters (solid line) and hurdlers (dashed line). VTC-F_0_ represent the maximal external force lower limbs could produce during a theoretical extension movement at null velocity; VTC-V_0_ represent to the maximal velocity at which lower limbs could extend during a theoretical extension under zero load.

The descriptive data of the horizontal and vertical mechanical F-V profile are shown in Table 1. Regarding the mechanical parameters during sprinting, there were significant differences between the female sprinters and hurdlers for HZT-Pmax (*t* = 3.67, *p* = 0.004, *d* = 2.1), HZT-V_0_ (*t* = 2.46, *p* = 0.03, *d* = 1.4) (Figure 1), and RFmax% (*t* = 3.1, *p* = 0.01, *d* = 1.8) (Figure 2) which were higher for the sprint athletes, while HZT-F_0_ (*t* = 2.0, *p* = 0.07, *d* = 1.15) tended to be higher for the sprint athletes and no differences were found in DRF% (*t* = −0.5, *p* = 0.62, *d* = 0.3). Regarding the mechanical parameters during the vertical squat jump trial, no differences were found between groups for VTC-F_0_ (*t* = 0.55, *p* = 0.5, *d* = 0.3), VTC-V_0_ (*t* = 0.78, *p* > = 0.45, *d* = 0.5) (Figure 3), and VTC-Pmax (*t* = 0.7, *p* = 0.5, *d* = 0.4).

**Table 1:** Descriptive data presented as mean ± standard deviation (SD), 95% confidence intervals, mean difference ± (SD), and 95% confidence intervals of the difference of the horizontal and vertical mechanical force-velocity profile displayed by event.

	**Mean (SD)**	**95% CI**	**Mean Difference (SD)**	**95% CI of the Difference**
**HZT-F**_**0**_ **(N·kg**^**−1**^**)**				
Sprinters	7.68 ± 0.45	7.21–8.15	0.69 ± 0.4	0.08–1.47
Hurdlers	6.99 ± 0.72	6.23–7.75		
**HZT-V**_**0**_ **(m·s**^**−1**^**)**				
Sprinters	9.37 ± 0.22*	9.13–9.60	0.45 ± 0.18*	0.04–0.86
Hurdlers	**8.91 ± 0.39**	8.50–9.32		
**HZT-Pmax (W·kg**^**−1**^**)**				
Sprinters	18.0 ± 1.12*	16.8–19.2	2.46 ± 0.6*	0.97–3.96
Hurdlers	**15.5 ± 1.20**	14.3–16.8		
**RFmax (%)**				
Sprinters	45.7 ± 1.27*	44.3–47.0	2.9 ± 0.9*	0.8–4.9
Hurdlers	**42.8 ± 1.86**	40.9–44.8		
**DRF (%)**				
Sprinters	−7.62 ± 0.48	−8.12– −7.12	−0.24 ± 0.4	−1.28–0.8
Hurdlers	−7.38 ± 0.10	−8.48– −6.29		
**VTC- F**_**0**_ **(N·kg**^**−1**^**)**				
Sprinters	39.2 ± 6.91	31.9–46.4	−2.1 ± 3.8	−10.6–6.45
Hurdlers	41.3 ± 6.36	34.6–47.9		
**VTC-V**_**0**_ **(m·s**^**−1**^**)**				
Sprinters	2.81 ± 0.69	2.08–3.53	0.25 ± 0.1	−0.45–0.96
Hurdlers	2.56 ± 0.35	2.19–2.93		
**VTC-Pmax (W·kg**^**−1**^**)**				
Sprinters	26.9 ± 5.09	21.6–32.3	1.75 ± 2.5	−3.83–7.33
Hurdlers	25.2 ± 3.40	21.6–28.8		

*HZT-F_0_, theoretical maximal horizontal force; HZT-V_0_, theoretical maximal horizontal velocity; HZT-Pmax, theoretical maximal horizontal power; RFmax, ratio of the horizontally-oriented component to the total ground reaction force; DRF, rate of decrease in ratio of force with increasing speed during sprint acceleration; VTC-F_0_, theoretical maximal vertical force; VTC-V_0_, theoretical maximal vertical velocity; VTC-Pmax, theoretical maximal vertical power*.

* *Significant differences from hurdlers (highlighted in bold): P < 0.05*.

## Discussion

The present study explored the mechanical properties and function of the lower-body through the F-V approach between high-level female sprinters and hurdlers. Supporting our hypothesis, sprinters were able to apply higher forward-oriented forces onto the ground during the acceleration phase and develop higher power outputs (*HZT-F*_0_*, RFmax, HZT-V*_0_*, HZT-Pmax*) than hurdlers with the magnitude of these differences being large.

The theoretical maximal velocity *(HZT-V*_0_*)* shows that female sprinters can keep producing horizontal force at higher velocities, which reflects a higher capability of lower limb to produce horizontal force at fast running speeds. This is also reflected in the fact that female sprinters have higher overall mechanical power output capabilities *(HZT-Pmax)* during the acceleration phase than female hurdlers. The ratio of force *(RF)* corresponds the ability to effectively orient the horizontal force at the first steps of the acceleration phase in relation to the total force produced. Female sprinters can apply more effectively the force developed by the lower limbs at low velocities, than hurdlers. It should be noted that *RF* is quantified by the first steps of the acceleration and is less representative of the entire acceleration phase. The ability to orient total force in the horizontal direction at each step to overall sprint acceleration phase *(DRF)* does not differ between female sprinters and hurdlers. Differences in the technique requirements between the events might be a reason for the differences in the mechanical abilities observed during sprinting between the two groups of athletes. In comparison to the technique requirements for the sprint start and the acceleration phase in short sprint events, the athletes of short sprint hurdles events after clearing the blocks tend to show a progressive increase in trunk angle at both touchdown and toe-off (Walker et al., 2019). The progression in trunk angle indicates a transition from the block start toward high velocity running, by producing a slightly larger total body vertical emphasis while allowing the trunk to extend more for the preparation into the first barrier (Walker et al., 2019). It can be hypothesized that through repetitive training hurdlers could adopt this technique while sprinting regardless of the presence of hurdles barriers affecting their ability to effectively apply the force onto the ground. This is in agreement with Kugler and Janshen (2010) who found that the further forward oriented ground reaction forces during acceleration, come together with further forward oriented body positions. However, it has to be mentioned that it is unknown whether specific hurdling training leads to the adoption of this specific technique while sprinting, regardless of the presence of hurdles barriers affecting so, their ability to effectively apply the force onto the ground. The present results also suggest that the ability to develop horizontal force during sprinting is not related with the ability of lower limbs to produce force, as obtained during jumping testing procedure, reflecting the lower limb neuromuscular properties. Nevertheless, in high-level to elite populations, horizontal force production during sprinting acceleration is likely less determined by the neuromuscular system capability to produce total force onto the ground as assessed through the vertical F-V-P profile. The differences in sprinting acceleration performance between sprinters and hurdlers may be more explained by differences in the mechanical effectiveness of force application between the events and especially by the ability to apply more effectively the force into the anteroposterior direction. These results are in agreement with previous studies that have revealed that high-level athletes are able to horizontally apply higher forces upon contact with the ground (Morin et al., 2011; Buchheit et al., 2014; Kawamori et al., 2014; Pantoja et al., 2016; Jiménez-Reyes et al., 2018b).

To our knowledge, this is the only study exploring the differences in horizontal and vertical F-V-P profile between high-level female sprinters and hurdlers during the competitive period of the season. The F-V-P approach is expected to be useful for both researchers and coaches in order to ensure a more specific, accurate, and comprehensive characterization of athletes' physical qualities toward better designed training programs. It will be of practical importance for track and field coaches to focus their training into improving the horizontal components of F-V-P profile, especially for the high-level female hurdlers. Females hurdlers clearing lower barrier heights, compared to men's 110-m hurdles event, and possibly, a specific training in order to achieve higher forward orientation of the produced force in the initial acceleration run could be leading into performance maximization. In addition, coaches should monitor their horizontal, and vertical FVP profile throughout the season in order to give emphasis in the components that each athlete should improve. Future research should investigate the differences in mechanical capabilities, the effectiveness of force application as well as to examine the kinetics and kinematics parameters to better understand the mechanisms behind the differences of the sprint-acceleration performance between high-level sprinters and hurdlers in order to design effective training programs.

The study has some limitations that must be addressed. The sample size of the present study is small and may reduce statistical power and increase the margin of error, which can affect the results. Furthermore, even though both sprinters and hurdlers are high-level athletes, differences in their performance level could be a reason for the different force dominant profile orientation in female sprinters compared to hurdlers and may be a derivative of a relatively small sample size. It should be noted, that the female athletes which involved in the current study were in the top national-level, and participated in the finals of the national championships in athletics competitions. Beyond that, the inverse dynamic model used in our study (Samozino et al., 2008, 2016; Morin et al., 2019) has limitations such as estimating the horizontal aerodynamic drag force from only stature, body mass and a fixed drag coefficient (Arsac and Locatelli, 2002), as well as having the assumption of a quasi-null center of mass vertical acceleration over the sprint-acceleration phase. The latter assumption is more pronounced when using starting blocks and less when starting from a three-point crouching position. Moreover, to ensure valid mechanical output computations based on velocity-time data, as obtained by a high-speed camera, it is crucial to correctly determine the frame corresponding to the start of the sprint which corresponds to the beginning of the force production. We consider the frame at which the athletes thumb left the ground from a three-point starting position as frame 0, which represent the moment of the force production. The same procedure was used in other studies as well (Romero-Franco et al., 2017; Morin and Samozino, 2018). However, we believe that the variables of the F-V and P-V mechanical profile were not affected by the methods used, since they were in agreement with other studies evaluating the same parameters.

## Conclusion

The main findings of the present study were that the high-level female sprinters are able to apply higher horizontally-oriented forces onto the ground during acceleration phase than the high-level female hurdlers. The practical applications of the present study support that the F-V-P profile is useful method for researchers and coaches in order to ensure a more specific, accurate and comprehensive characterization of high-level athletes' physical qualities in order to design effective training programs toward to performance maximization.

## Data Availability

The datasets generated for this study are available on request to the corresponding author.

## Ethics Statement

The studies involving human participants were reviewed and approved by Ethical Commitee of National and Kapodistrian University of Athens. The ethics committee waived the requirement of written informed consent for participation.

## Author Contributions

ISt and GP contributed to the conception and design of the study. All authors, ISt, ISm, AT, TE and GP participated in data base collection. ISt organized the database and performed the statistical analysis. ISt and ISm wrote the first draft of the manuscript. GP supervised the study. All authors contributed to manuscript revision, read and approved the submitted version.

### Conflict of Interest Statement

The authors declare that the research was conducted in the absence of any commercial or financial relationships that could be construed as a potential conflict of interest.
